# Revving the engine: PKB/AKT as a key regulator of cellular glucose metabolism

**DOI:** 10.3389/fphys.2023.1320964

**Published:** 2024-01-08

**Authors:** Xia Li, Shuying Hu, Yaoting Cai, Xuelian Liu, Jing Luo, Tao Wu

**Affiliations:** ^1^ General Practice Medical Center, West China Hospital, Sichuan University, Chengdu, China; ^2^ Department of Rehabilitation Medicine, West China Hospital, Sichuan University, Chengdu, China

**Keywords:** AKT, glucose uptake, glucose glycolysis, cancer, tricarboxylic acid cycle

## Abstract

Glucose metabolism is of critical importance for cell growth and proliferation, the disorders of which have been widely implicated in cancer progression. Glucose uptake is achieved differently by normal cells and cancer cells. Even in an aerobic environment, cancer cells tend to undergo metabolism through glycolysis rather than the oxidative phosphorylation pathway. Disordered metabolic syndrome is characterized by elevated levels of metabolites that can cause changes in the tumor microenvironment, thereby promoting tumor recurrence and metastasis. The activation of glycolysis-related proteins and transcription factors is involved in the regulation of cellular glucose metabolism. Changes in glucose metabolism activity are closely related to activation of protein kinase B (PKB/AKT). This review discusses recent findings on the regulation of glucose metabolism by AKT in tumors. Furthermore, the review summarizes the potential importance of AKT in the regulation of each process throughout glucose metabolism to provide a theoretical basis for AKT as a target for cancers.

## 1 Introduction

Glucose is the main energy source for most cells and is also an important substrate for biochemical reactions in cells. Glucose metabolism plays a key role in cell growth and proliferation of cells (Palm and Thompson, 2017; Shao et al., 2018). Intracellular glucose metabolism mainly includes the following steps: glucose uptake, glycogenesis and activation of the tricarboxylic acid cycle (TCA) (Han et al., 2016; Ye et al., 2022). A disorder in glucose metabolism can lead to metabolic diseases, such as obesity and type II diabetes (Lu et al., 2021), and cardiovascular disease (Stemmer et al., 2019; Poznyak et al., 2022). Furthermore, in an aerobic environment, cancer cells tend to undergo metabolism through glycolysis rather than the oxidative phosphorylation pathway (Abdel-Wahab et al., 2019). The phenomenon is called the Warburg effect (Wolf et al., 2010; Koppenol et al., 2011; Liberti and Locasale, 2016). Cancer cells can achieve this change in energy metabolism via increased activity of glucose transporters located on cancer cell membranes and enhanced activity of glycolytic enzymes (Zhou et al., 2008; Demasi et al., 2010). Thus, research evaluating mechanisms of glucose metabolism is helpful to identify feasible and effective therapy for the treatment of cancers.

AKT was originally identified and cloned from an AKT8 transforming retrovirus (Staal, 1987). Furthermore, AKT is also known as protein kinase B (PKB), considering its similarity with PKA and PKC (Coffer and Woodgett, 1991). Three subtypes of AKT are expressed has in mammals, namely AKT1, AKT2, and AKT3 (Vanhaesebroeck and Alessi, 2000; Siddika et al., 2022). AKT1 is activated and hyperphosphorylated in most human cancers by at two key regulatory sites (Thr308 and Ser473) (Xing et al., 2019). AKT1 is located in the cytoplasm and is essential for normal systemic growth (Cho et al., 2001). AKT2 is co-localized with mitochondria and is a major essential subtype involved in glucose metabolism (Toulany and Rodemann, 2015). AKT3 is located in the nucleus and nuclear membrane, and is crucial to achieve normal brain size (Easton et al., 2005). Phosphorylation of AKT can regulate the activity of downstream target proteins, which play an important role in controlling various important cellular functions such as cell apoptosis, glucose metabolism (Hua et al., 2021).

**FIGURE 1:**
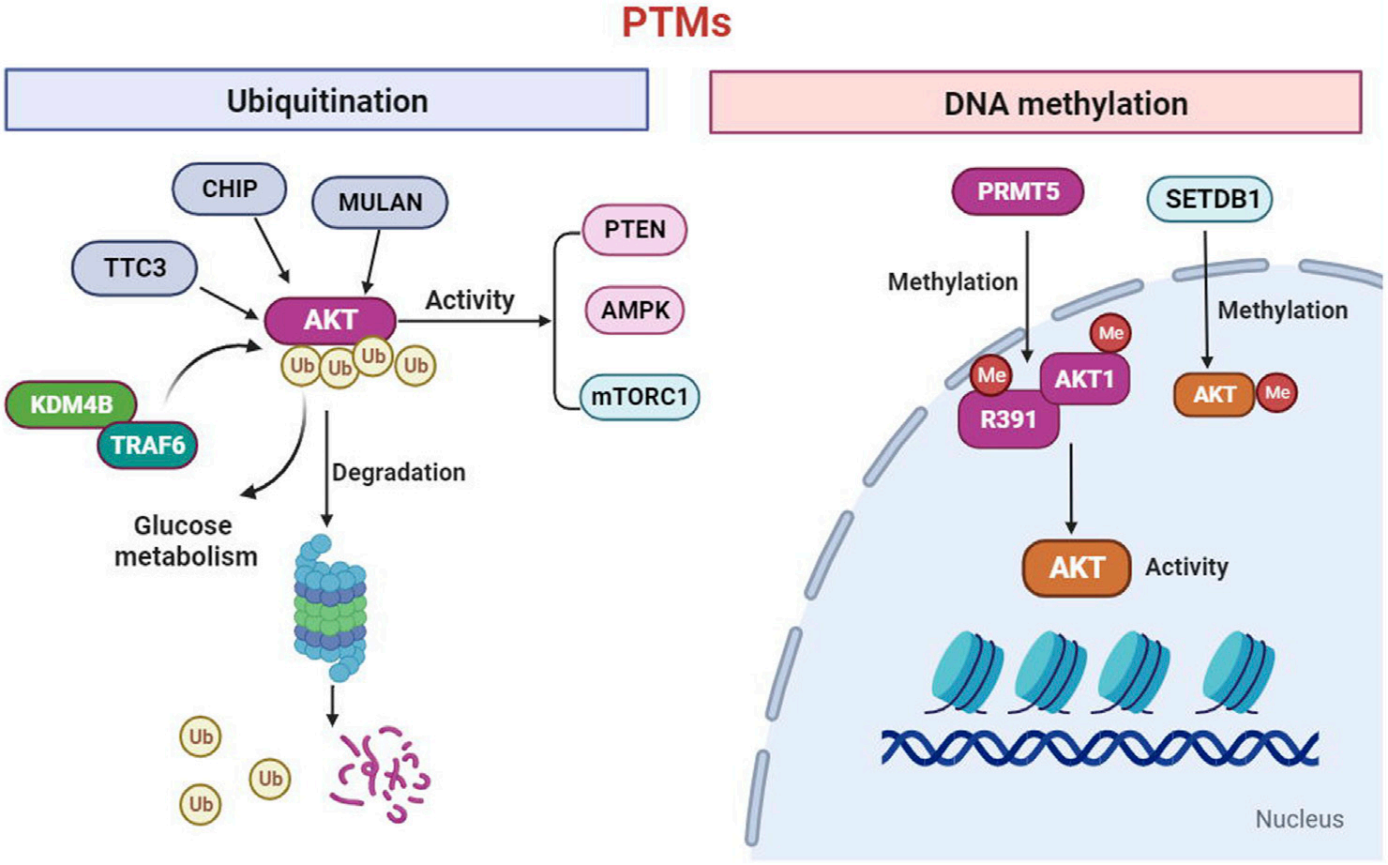
AKT can be modified through post-translational modifications (PTMs) including ubiquitination and methylation. The ubiquitination of AKT significantly regulates the activity of the mTORC1, AMPK and PTEN signaling. In addition, the CHIP, MULAN, Lysine Demethylase 4B (KDM4B) and TTC3 can ubiquitinate AKT and mediate its degradation. Furthermore, protein arginine methyltransferases 5(PRMT5) directly methylates AKT1-R391 to promote AKT activity. In addition, SETDB1-mediated methylation of AKT1 which promotes cell growth and glycolysis.

To date, it has been suggested that the AKT kinase can be modified though post-translational modifications (PTMs), including phosphorylation, ubiquitination, acetylation, methylation and hydroxylation (Guo et al., 2019). Phosphorylation has been extensively studied. A recent study extended the EPL technique to introduce a wider range of PTMs into AKT, an found that O-GlcNAcylation at Ser473 and phosphorylation at Tyr474 can activate the AKT activity toward peptide and protein substrates (Salguero et al., 2022). Ubiquitination, is a type of PTMs, the changed biological processes in tumor metabolism. The ubiquitination of AKT significantly regulates the activity of the mTORC1, AMPK and PTEN signaling pathways (Deng et al., 2020). In addition, the research determined that CHIP, MULAN, and TTC3 can ubiquitinate AKT and mediate its degradation (Guo, et al., 2019). The Lysine Demethylase 4B (KDM4B) could interact with TRAF6 and promote ubiquitination of AKT for activation, thereby promoting glucose metabolism in colorectal cancer cell (Li et al., 2020). DNA methylation is one of the most extensively studied and fully characterized epigenetic modifications. Emerging evidence demonstrated that protein arginine methyltransferases 5(PRMT5) directly methylates AKT1-R391 to promote AKT kinase activity (Yin et al., 2021). In addition, SETDB1-mediated methylation of AKT1 at K140 in cells, which promotes cell growth and glycolysis through AKT methylation (Figure Figure1). We should in-depth explore of the PTM functions of the AKT to develop novel strategies prevention of cancers.

Therefore, AKT is considered an effective target for cancer treatment. In this paper, we review the current knowledge about AKT signaling. We also discuss our current understanding of how AKT regulates glucose metabolism, including uptake, storage, and catabolism. This overview may shed new light on how to treat cancer by exploiting AKT as a molecular target for glucose metabolism.

## 2 AKT and glucose uptake

Glucose is the main form in which dietary sugars are made available to cells of the human body, and its breakdown is a major source of energy for almost all cells (Palm and Thompson, 2017). Glucose utilization begins with its uptake by cells through a glucose transporter. Glucose molecules are polar and bulky and cannot easily diffuse through the lipid membrane of cells (Navale and Paranjape, 2016). Glucose transporters comprise a wide range of membrane proteins that promote glucose transport across cell membranes (Han et al., 2018). Because glucose is an important source of energy for all life, these transporters are present in almost all cells. It mainly includes two types of transporters: sodium glucose linked transporters (SGLT) and facilitated diffusion glucose transporters (GLUTs) (Duelli and Kuschinsky, 2001; Hsu and Dzhagalov, 2019; Wang L. et al., 2022). GLUTs are integral membrane proteins containing 12 transmembrane helices with amino and carboxy termini exposed to the cytoplasmic side of the plasma membrane (Oka et al., 1990; Hruz and Mueckler, 2001; Chen and Russo, 2012). According to a model of alternative conformation, the GLUT protein transports glucose, expose an individual substrate binding site outside or inside of the cell. The binding of glucose to a site triggers conformational changes related to transport and glucose is released to the other side of the membrane (Hebert and Carruthers, 1992; Cloherty et al., 1995; Chen and Russo, 2012). Three subclasses of facilitative transporters have been identified (Bell et al., 1990; Thorens and Mueckler, 2010; Navale and Paranjape, 2016): Class I facilitative glucose transporters (GLUT1 to GLUT4) (Garvey et al., 1998; Godoy et al., 2006), Class Ⅱ facilitative glucose transporters (GLUT5, GLUT7, GLUT9, and GLUT11) (Li et al., 2004; Godoy, et al., 2006), and Class Ⅲ facilitative glucose transporters (GLUT6, GLUT8, GLUT10, GLUT12, and GLUT13) (Schmidt et al., 2009; Waller et al., 2013). The expression levels of GLUTs are regulated by PKB/AKT (Kupriyanova and Kandror, 1999; Kilic et al., 2007). After ingesting sugary substances, the blood glucose level is significantly increased, correspondingly, and insulin is secreted into the blood by the islets, which reduces the blood glucose level. In a physiological state, the GLUTs are sequestered into intracellular vesicles resulting in a low level of GLUTs at the cell surface (Bryant et al., 2002; Watson et al., 2004; Ramm and James, 2005). When blood sugar levels increase, insulin induces translocation of glucose transporter (e.g., GLUT1 and GLUT4) to the cell membrane, which activates glucose transport across the cell membrane, AKT is involved in this process (Guo et al., 2021). Knockdown of AKT or inhibition of AKT activation by LY294002 can negatively regulate the expression of GLUT1 on the plasma membrane (Lou et al., 2022). In contract AKT activation, induced by arsenic trioxide can promote cell proliferation by increasing GLUT1 expression that improves glucose uptake (Lou, et al., 2022). Furthermore, Zhang et al. found that activation of AKT/mTORC1/NF-κB signaling upregulated of GLUT1 transcription in nasopharyngeal carcinoma cells (Zhang et al., 2017). In contrast, inhibition of AKT activity by LY294002, a PI3 kinase (PI3K) inhibitor, abolishes insulin-induced induction of GLUT1 mRNA in Hepa1c1c7 cells (Barthel et al., 1999). Yu et al. determined that pretreatment with MK2206 (an AKT inhibitor) significantly reduced the effect of NRG-1β on glucose uptake and translocation of GLUT4 to the plasma membrane (Yu M. et al., 2022). In addition, hypoxia inducible factor-1α (HIF-1α), as a downstream target of AKT/mTORC1, has been reported to confer resistance to apoptosis under hypoxic conditions in rhabdomyosarcoma and Ewing sarcoma cells by increasing the expression of the glucose transporter GLUT1 (Fulda and Debatin, 2007; Kilic, et al., 2007). Based on these findings, we believe that AKT controls the transcription level of GLUTs by regulating transcription factors. In addition to affecting the transcription level of GLUTs, AKT has also been reported to be involved in translocation of GLUTs to the plasma membrane in response to insulin (DeFronzo et al., 2015). Studies have shown that AKT is associated with GLUT4-containing vesicles in 3T3-L1 adipocytes and rat cardiac ventricular muscle after insulin treatment (Kupriyanova and Kandror, 1999; Kessler et al., 2000; Hajduch et al., 2001), activated AKT phosphorylation is involved in the translocation to the plasma membrane of GLUT4 (Sasaki et al., 2009; Tan et al., 2012). The phosphorylation of the 160 kDa AKT substrate (AS160) can promote the transport of insulin-induced glucose transporter GLUT4 to the plasma membrane of adipocytes (Tan, et al., 2012). The phosphorylation of AS160 reverses the non-phosphorylated AS160 and GLUT4 vesicles, and promotes GLUT4 translocation (Tan, et al., 2012).

As a substrate for AKT, AS160 is phosphorylated by AKT at Ser-588 and Thr-642 sites in adipocytes (Kane et al., 2002). The role of AS160 in insulin-induced GLUT4 trafficking could be summarized as follows. Firstly, most GLUT4 is in intracellular storage vesicles (Bryant, et al., 2002; Watson, et al., 2004; Ramm and James, 2005). AS160 promotes hydrolysis of Rabs GTP to GDP on the GLUT4 storage vesicles so that Rabs in their inactive GDP-bound state inhibit GLUT4 exocytosis (Ishikura et al., 2007; Sano et al., 2007). Second, after phosphorylation under insulin stimulation, AS160 will bind to 14-3-3 proteins, which interact with phosphoserine or phosphothreonine residues in a variety of proteins to regulate several functions including subcellular redistribution, altered protein conformation, and impaired interaction with other proteins. AS160 dissociates from GLUT4 storage vesicles (Peck et al., 2006; Ramm et al., 2006; Geraghty et al., 2007). Thus, Rabs are loaded with GTP and promote the translocation of GLUT4 to the cell membrane, thus mediating the influx of glucose. Furthermore, TBC1D1 (tre-2/USP6, BUB2), phosphodylinositols (PI) and TXNIP (thioredoxin-interacting protein) also participate in AKT-regulated translocation of GLUT4 to the cell membrane (Taylor et al., 2008; Tan, et al., 2012). TBC1D1 is a member of the TBC1 Rab-GTPase family and may be involved in the regulation of GLUT4 translocation in skeletal muscle (An et al., 2010; Middelbeek et al., 2013; de Wendt et al., 2021). TBC1D1 and AS160 share 47% similarity, and have some structural features in common (Roach et al., 2007). TBC1D1 is also phosphorylated by AKT at the Thr-590 site and the role of TBC1D1 in insulin-induced GLUT4 translocation is similar to AS160 (Middelbeek, et al., 2013). PI3K is a relatively transient class of membrane phospholipids, which play a key role in the regulation of cell and organ functions, including signal transduction, ion channel gating, and vesicle movement (Tewari et al., 2022). PI3K is embedded in the lipid layer to regulate the formation, translocation, and anchoring of the GLUT4 vesicle (Chen et al., 2019; Liu et al., 2020). Phosphatidylinositol 3-phosphate 5-kinase (PIKfyve) is mainly responsible for catalyzing the phosphorylation of PI(3)P to PI(5)P and PI(3,5)P2 (Shisheva, 2008b; Rodgers et al., 2022). PI(3,5)P2 is present in GLUT4 vesicles and may regulate the transport of GLUT4 vesicles (Shisheva, 2008a). The study by Berwick et al. showed that serine 318 phosphorylation of PIKfyve (the substrate of AKT) promotes the trafficking of GLUT4 vesicles (Berwick et al., 2004). TXNIP is a type of α-arrestin protein that plays a role in the regulation of glucose and lipid metabolism (Bodnar et al., 2002; Sheth et al., 2005; Waldhart et al., 2017). Insulin stimulation can force TXNIP to separate from GLUT4 and inhibit the endocytosis of GLUT4, and ultimately improve glucose uptake (Liao et al., 2022). Waldhart et al. found that TXNIP acted as a substrate of AKT, activated AKT phosphorylation of TXNIP at Ser-308, and forced TXNIP to dissociate from the transporters (Waldhart, et al., 2017). Furthermore, TXNIP may inhibit AKT activity through a direct interaction with the PH domain of AKT under glucose stress (Huy et al., 2018). Therefore, insulin stimulation may induce glucose uptake in cells by activating AKT-mediated TXNIP phosphorylation, forcing TXNIP to dissociate from the transporters, thus inhibiting the endocytosis of the glucose transporter GLUT1 (Waldhart, et al., 2017).

**FIGURE 2:**
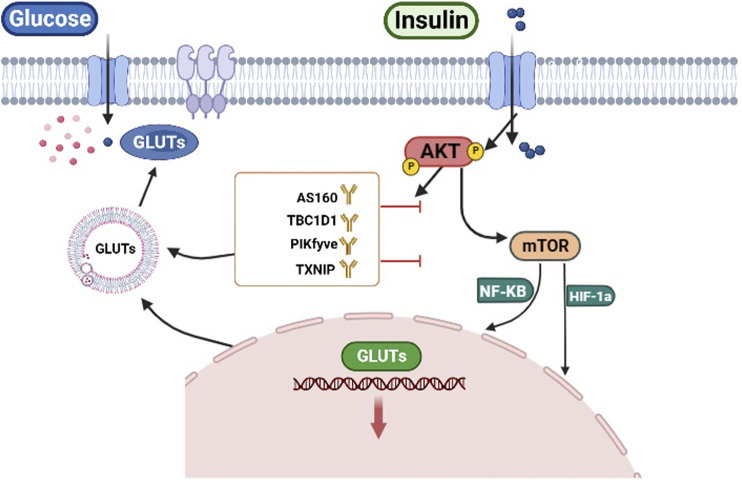
AKT regulates glucose uptake. AKT regulates the transcription of GLUTs by HIF-1α and NF-κB. Conversely AKT can directly phosphorylate targets (AS160, TBC1D1, PIKfyve, TXNIP) to regulate GLUTs trafficking.

In conclusion, AKT could control glucose uptake by regulating the transcription and localization of GLUTs to the cell membrane (Figure 2).

## 3 AKT and glycogenesis

Glycogen is a branched chain polymer of glucose, which can be used as the main energy storage of eukaryotes (Tang et al., 2020; Marr et al., 2022). Glycogenesis is a process of glycogen synthesis, in which glucose molecules bind to the glycogen chain for storage (Bandyopadhyay et al., 2022; Semrau et al., 2022). In mammals, glucose is deposited mainly on skeletal muscle and liver, and a little glucose is deposited on other tissues, including the kidneys, heart, fat, and brain (Roach et al., 2012). Glycogen synthase (GS) is a rate limiting enzyme in glycogenesis, and as such its activity is controlled by phosphorylation (inactivation) and dephosphorylation (activity) cycles (Li Q. et al., 2019; Marr, et al., 2022). Glycogen synthase kinase 3 (GSK-3) is an evolutionarily conserved intracellular serine/threonine kinase, which can inhibit GS and regulate glucose metabolism through phosphorylation (Wang N. et al., 2022). Under insulin stimulation, GSK3 is phosphorylated and inactivated by AKT, leading to inhibition of GS phosphorylation (Li T. et al., 2019). However, in primary hepatocytes treated with GlcN, exposure to LY294002 or MK2206 abolished the activity of irisin in promoting GSK3 phosphorylation and reducing GS phosphorylation (Liu H. et al., 2015). Furthermore, many cell signaling pathways have been shown to be related to signal transduction, such as the PI3K/Akt, Wnt, MAPK, and TGF-β signaling pathways (McCubrey et al., 2014; Ngeow et al., 2018; Duda et al., 2020; Papadopoli et al., 2021). Two GSK-3 isoforms exist in mammalian cell, GSK-3α and GSK-3β (Beurel et al., 2015; Okada et al., 2004; von Wilamowitz-Moellendorff et al., 2013), and inhibition of GSK-3 activity by insulin may be due to AKT-catalyzed phosphorylation of an N-terminal serine residue (Ser-21 in GSK-3α and Ser-9 in GSK-3β) (Cross et al., 1995; Li et al., 2010). Studies have shown that PI3K/AKT/GSK3β signaling pathways mediate glycogenesis in the liver (Valicherla et al., 2019). Therefore, after insulin activates AKT, AKT may in turn regulate blood glucose levels by promoting glucose uptake by regulating GLUTs, although AKT promotes glycogen synthesis, or by inhibiting GSK3 activity to activate GS activity. In conclusion, AKT could control glycogen synthesis by regulating GSK3 activity.

## 4 AKT and glycolysis

After glucose is transported into cells, it is further catabolized to pyruvate by glycolysis (Ganapathy-Kanniappan and Geschwind, 2013; Liu et al., 2021). Multiple enzymes are involved in this process, and AKT regulates these enzymes in a variety of ways. AKT could regulate the transcriptions of glycolysis enzymes via the activation of several transcription factors, such as FoxO, c-Myc, and HIF (Du et al., 2014; Daher et al., 2021; Pan et al., 2022).

### 4.1 FoxOs

The FoxO Forkhead transcription factors are highly conserved, and are regulated and controlled by the insulin signaling pathway (Nakae et al., 2008; Lee and Dong, 2017; Bhardwaj et al., 2021). FoxOs consist of FoxO1, FoxO3, FoxO4, and FoxO6, which regulate a series of cellular processes including proliferation, apoptosis, and cell metabolism (Tzivion et al., 2011; Klotz et al., 2015). FoxOs play an important role in glycogenolysis by insulin signaling and are located downstream of AKT (Sergi et al., 2019). The transcriptional functions of FoxOs are regulated by their nucleocytoplasmic transport. Under insulin stimulation, AKT is activated and phosphorylates FoxOs, which leads to the export of FoxOs to the cytoplasm (Biggs et al., 1999; Brunet et al., 1999; Kops and Burgering, 1999; Kim et al., 2018). Furthermore. PKB/Akt-dependent phosphorylation of FoxOs transcription factors favors their sequestration in complex with 14-3-3 proteins in the cytoplasm (Rena et al., 2001). FoxO1, FoxO3 and FoxO4 contain three AKT phosphorylation sites, respectively, while FoxO6 contains two. For example, the three sites of murine FoxO1 are Thr24, Ser253 and Ser316 (Nakae, et al., 2008). In murine hematopoietic stem cells (HSCs), the conditional deletion of cited2 resulted in decreased glycolysis; however, inhibition of PI3/Akt (LY294002) partially rescued FoxO phosphorylation and promoted glycolysis (Du, et al., 2014). The transcription of hexokinase (HK) and pyruvate kinase (PK) are repressed by FoxOs activation (Shiau et al., 2022), thus AKT promotes transcription of glycolytic enzymes by inhibiting the nuclear localization of FoxOs (Haeusler et al., 2010; Zhang et al., 2012; Knight et al., 2014; Lien et al., 2016).

### 4.2 c-Myc

c-Myc is a key transcription factor and plays an important role in many physiological processes such as cell growth, apoptosis, and energy metabolism (Cramer and Schmitt, 2016; Allen-Petersen and Sears, 2019). Multiple regulatory mechanisms are mediated by AKT to regulate c-Myc activity (Cramer and Schmitt, 2016). For example, AKT controls the stability of the c-Myc protein by GSK3β, which in turn phosphorylates c-Myc at Thr58 and promotes its subsequent ubiquitination by the SCF ubiquitin ligase (Gregory et al., 2003; Welcker et al., 2004). AKT inactivation could result in down-regulation of the post-translational expression of Pim-1 and c-Myc protein. Post-translational down-regulation of c-Myc has been reported to occur through T58 phosphorylation caused by GSK-3 inactivation of GSK-3β (Scarpa et al., 2021). c-Myc is translocated into the nucleus to upregulate the transcription of glycolysis enzymes, including HK, G-6-PI and phosphofructokinase (Yang et al., 2018; Yu Q. et al., 2022). In hepatocellular carcinoma cells, the AKT inhibitor (LY294002) was found to reduce the expression of c-Myc, HK2 and PKM2, further inhibiting glycolysis (Shin et al., 2021). Thus, AKT promotes the transcription of glycolysis enzymes by c-Myc.

### 4.3 HIF

The HIF family is activated in response to hypoxia and consists of three members, HIF1, HIF2, and HIF3 (Cramer and Schmitt, 2016; Catrina and Zheng, 2021; Knutson et al., 2021). HIFs direct glucose away from oxidative phosphorylation toward glycolysis and lactate production (Kierans and Taylor, 2021; Knutson, et al., 2021). The PI3K/AKT pathway has been demonstrated to be involved in regulating HIF-1α expression and transcriptional under hypoxic conditions (Xie et al., 2019; Kierans and Taylor, 2021). In esophageal carcinoma cell lines, hypoxic conditions result in a upregulation of HIF-1α protein and glycolytic enzyme expression, whose expression can be reversed using the PI3K/AKT signal inhibitor wortmannin (Zeng et al., 2016; Kierans and Taylor, 2021). Although the above research indicates that the PI3K/AKT signaling pathway can affect the stability of HIF-1 to regulate glucose metabolism, further research is needed to determine the extent to which the PI3K/AKT signaling pathway is involved in HIF-dependent glucose metabolism.

### 4.4 Glycolytic enzymes

In addition to regulating the transcription of glycolytic enzymes, AKT is involved in the regulation of the phosphorylation status of multiple glycolytic enzymes and their activity. HK catalyzes the first step of glycolysis by phosphorylating glucose to form glucose 6-phosphate (G-6-P) (Rathmell et al., 2003; Deshmukh et al., 2016). AKT promotes the association of HK1 and HK2 with mitochondria to increase HK activity and directs G6P toward glycolysis (Gottlob et al., 2001; John et al., 2011). AKT directly phosphorylates HK2 at Thr473 to promote mitochondrial association of HK2 mitochondrial association (Yu et al., 2019). AKT also inhibits GSK-3β and indirectly promotes mitochondrial binding to HK2, while also phosphorylating VDAC and inhibiting its ability to bind to HK2 (Pastorino et al., 2005; Cramer and Schmitt, 2016). In the glycolytic pathway, phosphofructokinase 1 (PFK1) catalyzes a rate-limiting step of glycolysis by converting fructose 6-phosphate to fructose 1,6-bisphosphate (Li et al., 2017; Feng et al., 2020). The platelet isoform PFK1 (PFKP) is the predominant isoform of PFK1 (Lee et al., 2017). Lee et al. showed that AKT phosphorylates PFKP at Ser386, and this phosphorylation inhibits PFKP degradation of PFKP (Lee, et al., 2017). Additionally, AKT can phosphorylate the glycolytic activator (PFK-2/FBPase-2), resulting in increased PFK2 activity (Li, et al., 2017; Simon-Molas et al., 2018). Glyceraldehyde-3-phosphate dehydrogenase (GAPDH) is a glycolytic enzyme found in almost all tissues (Tristan et al., 2011; Zhang et al., 2015). AKT may be involved in the regulation of the enzymatic activity of GAPDH. In the HuCCT1 tumor, GAPDH was demonstrated to be a downstream signaling molecule of the Hic-5/Sec/AKT signaling pathway that participated in HuCCT1 cell migration (Wu WS. et al., 2022). However, the specific mechanism involved in AKT regulation of GAPDH activity needs further study. Phosphate glycerate kinase (PGK) catalyzes the reversible reaction of 1,3-diphosphate glyceride (1,3-BPG) with ADP in the glycolytic pathway, forming 3-phosphate glyceride (3-PG) adenosine triphosphate (ATP) (Wang et al., 2007; Wang et al., 2015). A recent study demonstrated that increased expression of PGK1 promoted glycolysis and enhanced the progression of epithelial mesenchymal transition (EMT) in oral squamous cell carcinoma (OSCC) cells (Zhang et al., 2020; Marcucci and Rumio, 2022). PGK1 activity can be inhibited by acetylation at lysine 220 (Wang, et al., 2015). HDAC3 is responsible for the deacetylation of PGK1 to increase PGK1 activity. The AKT/mTORC1 pathway controls HDAC3 S424 phosphorylation to regulate its activity. Hence, AKT indirectly activates PGK1 by HDAC3-mediated PGK1 deacetylation. Pyruvate kinase (PK) is the enzyme that catalyzes the glycolysis process, and consists of four isozymes in vertebrates: PKL, PKR, PKM1, and PKM2 (Chaneton and Gottlieb, 2012). Pyruvate kinase muscle type 2 (PKM2) has been shown to promote cancer cell metabolism and growth (Li Q. et al, 2019). PKM2 is regulated by phosphorylation, which leads to the translocation from the cytoplasm to nucleus (Luo et al., 2011; Yang et al., 2012). PKM2 activity may be inhibited after glycolysis phosphorylation. For example, Hitosugi et al. demonstrated that the fibroblast growth factor receptor 1 (FGFR1) induced the phosphorylation of PKM2 at Tyr105, leading to a decrease in lactate production (Park et al., 2016). Meanwhile, PKM2 has also been reported to be a substrate of AKT (Hitosugi et al., 2009). Park et al. showed that AKT can directly interact with PKM2 and phosphorylate it at Ser202, which is crucial for nuclear translocation of the PKM2 protein under stimulation by insulin-like growth factor 1 (IGF-1) (Park, et al., 2016). The increase in nuclear localization of PKM2 is consistent with the increased expression of GLUT1, HK2, and HIF-1α and glucose entrapment (Salani et al., 2015). This suggests that after transfer of PKM2 to the nucleus, AKT is involved in the regulation of glucose intake.

**FIGURE 3:**
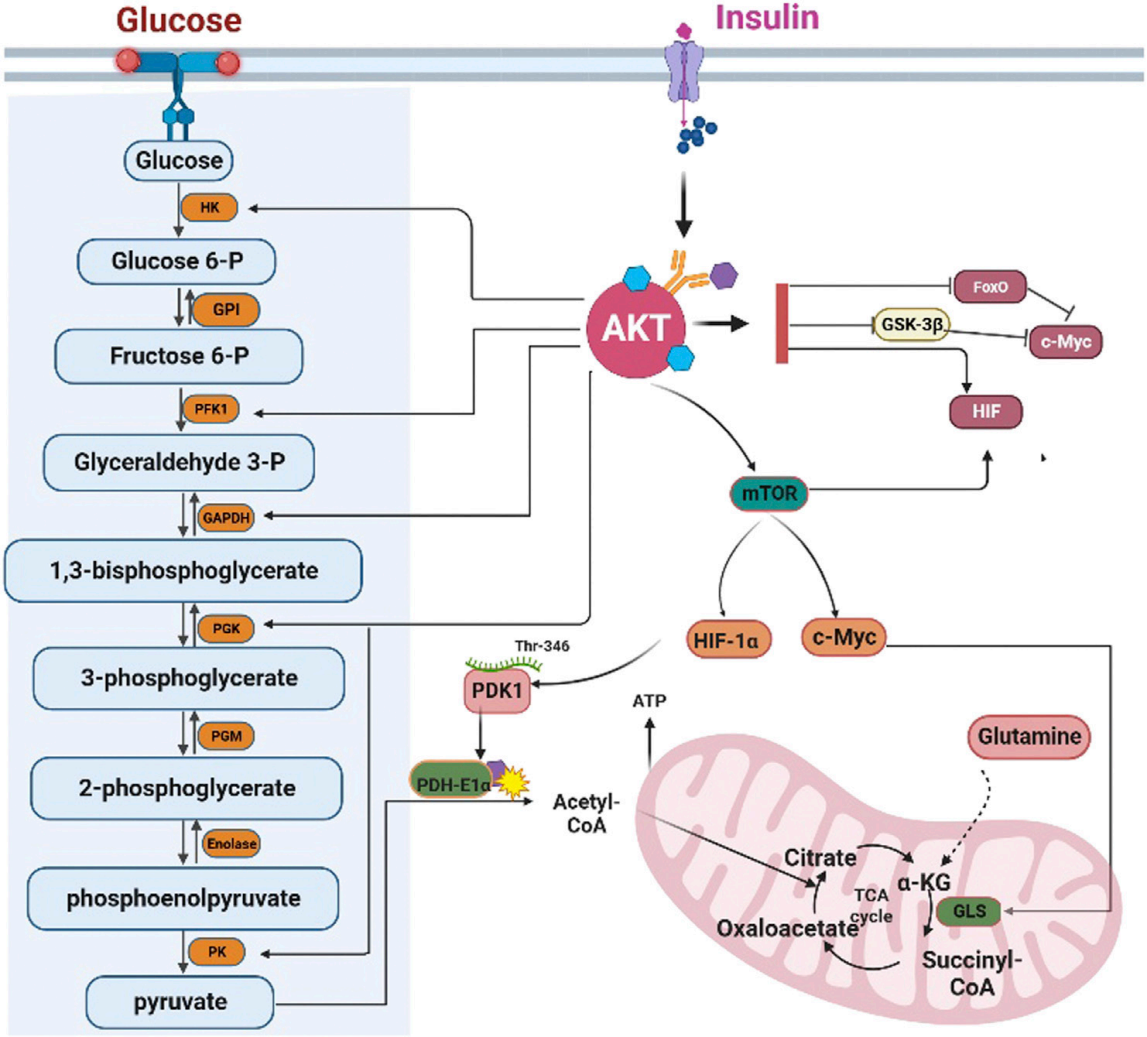
AKT regulates glycolysis and the tricarboxylic acid cycle. AKT regulates the transcription of glycolysis enzymes by FoxOs, c-Myc, and HIF-1α. However, AKT can directly phosphorylate the glycolytic enzymes HK, PFK1, GAPDH, PGK, and PK. HK: Hexokinase; GPI: Glucose 6-phosphate isomerase; PFK1 phosphofructokinase 1; TPI triose phosphate isomerase; GAPDH, Glyceraldehyde 3-phosphate dehydrogenase; PGK: phosphoglycerate kinase; PGM: Phosphoglycerate mutase; PK: Pyruvate kinase. AKT can inhibit the influx of acetyl-CoA into the TCA cycle by inhibiting PDH-E1αactivity. Furthermore, AKT can promote the conversion of glutamine into α-ketoglutarate to compensate for the depletion of the intermediates of the TCA cycle. GLS: Glutaminase, α-KG: α-ketoglutarate.

In conclusion, AKT could control glycolysis by regulating the activity of multiple glycolytic enzymes (Figure 3).

## 5 AKT and the tricarboxylic acid cycle

The tricarboxylic acid (TCA) cycle is a significant link in glucose metabolism. Pyruvate is transferred into mitochondria and further converted into acetyl-CoA, which is oxidized to ATP and carbon dioxide through TCA cycle (Bowtell et al., 2007; Arnold et al., 2022). Pyruvate from glycolysis enters the TCA cycle through the action of the pyruvate dehydrogenase complex (PDC), which is a compound of three enzymes that converts pyruvate into acetyl-CoA through decarboxylation of pyruvate (Patel et al., 2014; Golias et al., 2019; Lima-Silva et al., 2022). Pyruvate dehydrogenase (PDH) E1α phosphorylation of subunits can inhibit PDC activity (Patel and Korotchkina, 2006; Cerniglia et al., 2015; Sharma et al., 2019). Therefore, phosphorylation of PDH-E1α phosphorylation suppresses the entry of pyruvate into the TCA. Phosphorylation of PDH-E1α is regulated by pyruvate dehydrogenase kinase (PDK) (Sharma, et al., 2019). AKT can regulate PDK activity to further control PDH-E1α activity. As mentioned above, the HIF transcription factors are regulated by AKT/mTORC1 signaling and Kim et al. demonstrated that PDK1 was a direct HIF-1α target gene (Kim et al., 2006); thus, AKT can indirectly regulate the PDH-E1α activity via the HIF-1α/PDK1 axis. Conversely, AKT can phosphorylate PDK1 at Thr-346 during hypoxia, inactivating pyruvate dehydrogenase (Chae et al., 2016). Based on the above, AKT has an impact the influx of acetyl-CoA into the TCA cycle by inhibiting PDC activity.

The TCA cycle is a key metabolic pathway that connects carbohydrate, fat, and protein metabolism. The enzymes in the cyclic reaction can completely oxidize acetyl-CoA into two molecules each of carbon dioxide and water (Liu TY. et al., 2015; Ryan et al., 2021). 3-Methyladenine (3-MA) could lead to inhibition of AKT phosphorylation and inhibition of AKT phosphorylation can reduce the activities of TCA enzymes (Shaerzadeh et al., 2014). Activities of five enzymes in the TCA cycle including citrate synthase, aconitase, fumarase, a-ketoglutarate dehydrogenase, and malate dehydrogenase were reduced in the presence of 3-methyladenine, which inhibits the PI3K/AKT signaling pathway (Shaerzadeh, et al., 2014). However, the specific mechanism of AKT that regulates the activity of TCA enzymes needs further study. The TCA cycle provides intermediates for biosynthetic reactions such as amino acids, nucleotides, and lipids (Cramer and Schmitt, 2016). Due to the metabolic transformation of cancer cells into aerobic glycolysis, cancer cells must coordinate mechanisms to compensate for the consumption of intermediates of the TCA cycle intermediates (Cramer and Schmitt, 2016). In one major pathway, glutamine is converted into α-ketoglutarate (α-KG) and feeds into the TCA cycle (Fan et al., 2013; Hensley et al., 2013). c-Myc is a downstream gene of the AKT/mTORC1 pathway, and can up-regulate the transcription of glutamine transporters ASCT2 and SN2 to stimulate glutamine uptake (Wise et al., 2008; Li and Simon, 2013). The rate-limiting step for glutamine utilization is regulated by the enzyme glutaminase (GLS) (Mukha et al., 2021). GLS transforms glutamine into glutamate to support the TCA together with redox and epigenetic reactions (Johnson et al., 2018). Some studies have shown that GLS is a c-Myc target gene (Wise, et al., 2008; Gao et al., 2009; Li and Simon, 2013). Thus, the AKT/mTORC1 pathway can regulate the TCA cycle by promoting the conversion of glutamine into α-KG.

Overall, AKT controls the TCA cycle by regulating the influx of acetyl-CoA and α-KG into the TCA cycle.

## 6 Conclusion

In recent decades, there has been renewed interest in explaining how metabolism is altered in metabolic syndromes and cancers based on observations of signal transduction pathway molecules that regulate glucose metabolism (Cramer and Schmitt, 2016). The AKT signaling pathway is involved in the regulation of multiple processes, and researchers have recognized the importance of regulation and function of AKT signaling. Undoubtedly, AKT signaling is closely related to glucose metabolism and its manipulation and activation of its downstream molecules represent promising therapeutic targets for the treatment of metabolic syndromes and cancers. Furthermore, the AKT signaling pathway can be activated due to the expression of certain oncogenes or the omission of certain tumor suppressor genes (Ma et al., 2022). The overexpression of AKT has been demonstrated in many cancers, including ovarian cancer, lung cancer, and pancreatic cancer. At the same time, abnormal activation of AKT is related to increased cancer cell proliferation, survival, and drug resistance (Jacobsen et al., 2017; Ediriweera et al., 2019; Wu Y. et al., 2022; Gu et al., 2022).

Various Akt inhibitors with potential inhibitory functions are being investigated in preclinical studies. AKT inhibitors are classified according to their inhibition mechanisms and chemical scaffolds, and are classified as ATP competitive inhibitors (GSK690693, GDC0068 and AZD5363), allosteric inhibitors (pyranonaphthoquinone lactones), and irreversible inhibitors. Currently, AKT is considered a potential target for cancer treatment and prevention, and some AKT inhibitors have been clinically approved for the treatment of cancer (Chugh et al., 2008). The AKT inhibitor MK-2206 and LY2940002 has been shown to block glucose metabolism. Furthermore, the administration of competitive ATP inhibitors (GSK690693) in mice can significantly inhibit glycogen synthesis and peripheral glucose uptake (Crouthamel et al., 2009). Furthermore, many AKT inhibitors, including patasertib and capivasertib, exert anticancer activity in preclinical studies, and clinical trials are being advanced (Hua, et al., 2021). However, it has not been reported whether all AKT inhibitors are involved in regulating and inhibiting glucose metabolism pathways, except for MK-2206, LY2940002 and GSK690693. Thus, further in-depth research is warranted to investigate the regulatory effects of currently reported AKT inhibitors and to develop new inhibitors. AKT provides a novel target and the molecular mechanisms of AKT regulation in glucose metabolism should be exploited to improve cancer treatment. Small interfering RNA (siRNA) directed against AKT confirmed its impact on glucose metabolism. In HeLa lines, AKT-siRNA enhanced glucose uptake and HK2 expression, suggesting that inhibition of AKT may increase glucose uptake by detaching HK2 from the mitochondria (Neary and Pastorino 2013). Non-etheless, there is great heterogeneity in the energy metabolism of cancer cells, and even the same malignant tumor may exhibit differences in metabolism, which presents challenges to energy metabolism as a target for treatment. To avoid this phenomenon, AKT-specific inhibitors could be used in combination with metabolic target inhibitors, or dual inhibition can be achieved focusing on these targets to achieve better therapeutic properties (Song et al., 2019).

## Abbreviations

PKB/AKT, Protein kinase B; TCA, Tricarboxylic acid cycle; SGLT, Sodium glucose linked transporters; GLUTs, Glucose transporters; GSK-3, Glycogen synthase kinase 3; HK, Hexokinase; PK, Pyruvate kinase; G-6-P, Glucose 6-phosphate; PFK1, Phosphofructokinase 1; PGK, Phosphate glycerate kinase; ATP, Adenosine triphosphate; EMT, Epithelial mesenchymal transition; PDC, Pyruvate dehydrogenase complex; PDK, Pyruvate dehydrogenase kinase; 3-MA, 3-Methyladenine; PGM, phosphoglycerate mutase; PTMS, post-translational modifications; KDM4B, The Lysine Demethylase 4B; PRMTS, Protein arginine methyltransferases 5.
